# T1 values by conservative septal postprocessing approach are superior in relating to the interstitial myocardial fibrosis: findings from patients with severe aortic stenosis

**DOI:** 10.1186/1532-429X-17-S1-P49

**Published:** 2015-02-03

**Authors:** Nicholas Child, May Lin Yap, Darius Dabir, Toby Rogers, Gonca Suna, banher sandhu, David M Higgins, Manuel Mayr, Eike Nagel, Valentina Puntmann

**Affiliations:** 1 King's College London, London, UK; 2 Philips Healthcare, Guildford, UK

## Background

Accumulation of diffuse interstitial myocardial fibrosis in severe aortic stenosis relates to left ventricular (LV) functional impairment and determines the postoperative outcome in these patients. T1 mapping has been proposed for non-invasive quantification of diffuse interstitial fibrosis. We examined associations between T1 values and collagen volume fraction from endomyocardial biopsies from patients with severe aortic stenosis. We compared whether these relationships differ with respect to the post-processing approach.

## Methods

Ten patients (mean age 70 years, 6 male) with isolated severe aortic stenosis and eligible for aortic valve replacement surgery underwent clinical cardiovascular magnetic resonance study at 3 clinical scanner. A mid- chamber myocardial biopsy was obtained from the left ventricular (LV) septum at the time of surgery and stained for collagen volume fraction (CVF) using Mason Trichrome technique and ImageJ analysis.

T1 mapping was performed using MOLLI (3(3)3(3)5)) sequence prior to and 15 minutes after IV administration of 0.2 mmol/kg of gadobutrol. T1 measurements were performed using both a conservative septal ROI and the entire coverage of midventricular short-axis (SAX) slice. Areas of overt LGE were excluded from the analysis. T1 values were compared to fourteen age-matched healthy control subjects and correlated with CVF.

## Results

Compared to controls, there were no significant differences in cardiac volumes and global systolic function. Patients had raised LV mass and wall thickness (p<0.01), and mean native T1 values (Table 1). Four patients showed intramyocardial late gadolinium enhancement, 3 in inferolateral wall and one in anterior-septal segment. In patients mean CVF measured 21.3±13%. T1 values showed significantly stronger association with CVF when sampled by septal ROI. The association was the strongest for native T1 (p<0.001).

**Table 1:** 

	Controls (n=14)	AS patients (n=10)	Controls (n=14)	AS patients (n=10)
	Septal ROI		SAX ROI	

Native T1	1050±21	1109±27**	1042±23	1098±49*

Postcontrast T1	453±31	439±48	462±46	443±52

Lambda	0.46±0.03	0.48±0.06	0.46±0.07	0.48±0.1

ECV	0.26±0.04	0.29±0.06	0.27±0.08	0.29±0.09

Pearson correlation with collagen volume fraction (r, p-value)				

Native T1		0.59**		0.36*

Postcontrast T1		-0.31*		-0.27

Lambda		0.42*		0.29*

ECV		0.42*		0.24

				

* p<0.05, **p<0.01

## Conclusions

Our results inform on the ability of myocardial T1 indices to relate to the underlying interstitial myocardial fibrosis with respect to the post-processing approach. We show that interstitial fibrosis was best correlated with septal native T1 values, indicating that the differences in hybrid measures are likely driven by the signal of native T1. Our findings suggest that native T1 may represent the optimal index and a candidate technique for supporting the clinical use of T1 mapping in non-invasive measurement of diffuse fibrosis.

## Funding

Department of Health via the National Institute for Health Research (NIHR) comprehensive Biomedical Research Centre award to Guy's & St Thomas' NHS Foundation Trust in partnership with King's College London and King's College Hospital National Health Service Foundation Trust, and British Heart Foundation Research Centre of Excellence Award. King's Health Partners via MRC Confidence in Concept Grant.

